# Insights into coordination and ligand trends of lanthanide complexes from the Cambridge Structural Database

**DOI:** 10.1038/s41598-024-62074-3

**Published:** 2024-05-17

**Authors:** Shicheng Li, Santa Jansone-Popova, De-en Jiang

**Affiliations:** 1 Department of Chemical and Biomolecular Engineering, Vanderbilt University, Nashville, TN 37235 USA; 2 Chemical Sciences Division, Oak Ridge National Laboratory, Oak Ridge, TN 37831 USA

**Keywords:** Cheminformatics, Coordination chemistry

## Abstract

Understanding lanthanide coordination chemistry can help develop new ligands for more efficient separation of lanthanides for critical materials needs. The Cambridge Structural Database (CSD) contains tens of thousands of single crystal structures of lanthanide complexes that can serve as a training ground for both fundamental chemical insights and future machine learning and generative artificial intelligence models. This work aims to understand the currently available structures of lanthanide complexes in CSD by analyzing the coordination shell, donor types, and ligand types, from the perspective of rare-earth element (REE) separations. We obtain four sets of lanthanide complexes from CSD: Subset 1, all Ln-containing complexes (49472 structures); Subset 2, mononuclear Ln complexes (27858 structures); Subset 3, mononuclear Ln complexes without cyclopentadienyl ligands (Cp) (26156 structures); Subset 4, Ln complexes with at least one 1,10-phenanthroline (phen) or its derivative as a coordinating ligand (2226 structures). The subsequent analysis of lanthanide complexes in these subsets examines the trends in coordination numbers and first shell distances as well as identifies and characterizes the ligands and donor groups. In addition, examples of Ln-complexes with commercially available complexants and phen-based ligands are interrogated in detail. This systematic investigation lays the groundwork for future data-driven ligand designs for REE separations based on the structural insights into the lanthanide coordination chemistry.

## Introduction

Rare earth elements (REEs), encompassing lanthanides, yttrium, and scandium, find extensive utility in numerous technological applications, including magnets, superconductors, batteries, display devices, fluorescent materials, and catalytic converters^1^. Although they are relatively abundant in Earth’s crust, REEs are rarely found in concentrated and economically exploitable forms in nature; they also have similar chemical properties, making their separations difficult but necessary^2,3^. The most commonly used process for REE separations is solvent extraction^4^, which has been commercially practiced in large scale since 1960s. Prior to this, methods such as fractional crystallization and chromatographic separation were utilized to separate individual lanthanides. During the solvent extraction process, an organic extractant or ligand forms complexes with the REE ions; the selective transfer of ions from the aqueous phase to the non-aqueous phase is determined by the stability of the formed complex^5,6^.

The critical materials need for magnets in electric motors from the rapid rise in adoption of elective vehicles has been driving the recent resurgence in fundamental research of REE separations chemistry. Researchers have recently explored novel organic ligand design^7–12^, spectroscopic characterization of the coordination environment^13,14^, redox/photo-stimulated processes^15^, ionic liquids media^16–20^, biomolecule ligands^21,22^, as well as novel materials^23^. At the heart of these recent developments is the quest for a deeper understanding of the coordination chemistry of lanthanides^24,25^. Ln ions are highly adaptable to a diverse array of coordination environments^26^ and the single-crystal X-ray crystallography is still the most powerful tool to reveal the atomistic details of such coordination environments.

The Cambridge Structural Database (CSD), the world’s largest database of small-molecule organic and metal–organic crystal structures, contains tens of thousands of lanthanide complexes^27^. With rapid advancements in data-driven machine learning (ML) and artificial intelligence (AI)^28^, one hopes to leverage the CSD to generate and design new ligands for the desired coordination environment that can facilitate efficient separations of REEs. This is on one hand inspired by the huge success of AlphaFold in protein structure prediction^29^, trained on the Protein Data Bank (PDB) of about 100,000 unique protein structures determined mainly by single-crystal X-ray crystallography. On the other hand, researchers have been devising algorithms to automate the creation of the complex structures^30,31^. Hence, there is a great opportunity in learning from the CSD to create new ligands and new complex structures, as has been demonstrated recently^32^.

Toward the goal of structure-based, data-driven design of ligands for REE separations, we think that the first step is to have a statistical understanding of the currently available data in CSD. This approach had been previously pursued by Huang and coworkers for 1391 complexes published between 1935 and 1995, which prompted the present work to also take advantage of the progress in the past 30 years^33^. Hence, the goal of the present work is to identify all Ln complexes in CSD up to date, create subsets of interest, and conduct an in-depth analysis of their coordination chemistry, which will lay a foundation for further ML and generative AI approaches toward ligand design for REE separations.

## Methods

Using the CSD Python-based applications programming interface (API)^27,34^, we have developed task-specific scripts to search and analyze downloaded CSD structures. Each script entailed the retrieval of structures with three-dimensional structural information from the CSD and the extraction of chemical information from these structures. The investigation of the coordination number of lanthanides primarily relied on analyzing the first coordination shell. Consequently, the first script was designed to capture the first shell from the original structure. The second script was devised to explore the distribution of elements or donor groups within the first shell. Building upon the core components established in the first script, the primary objective of this script was to generate a matrix capable of recording the occurrences of different atoms within the first coordination shell. The third script aimed to analyze the type of ligands binding to the metal center. A module was developed to recognize ligands and differentiate them between organic and inorganic ligands: organic ligands are identified by possessing a carbon chain; inorganic ligands are categorized by small molecules (like water, nitrate, chloride) and polyoxometalates (POMs). The fourth script was designed to extract lanthanide complexes with phenanthroline and its derivatives as ligands. A module was developed to identify the complexes with the phenanthroline ligand itself. A few other scripts were developed as well for recognizing specific ligands. Further analysis of ligand types and phenanthroline derivatives was conducted manually. Each cyclopentadienyl ligand’s contribution to the coordination number was counted as three^33^. See the data availability statement for access to the datasets and our scripts.

## Results and discussion

### Available complex structures across the lanthanide series

**Figure 1:**
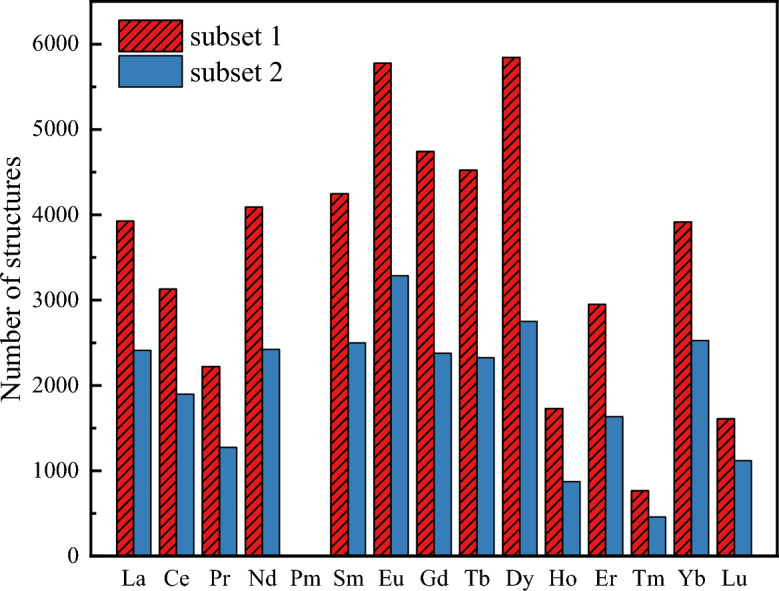
Distribution of the 49,472 crystal structures of Subset 1 (all Ln complexes) and the 27,858 crystal structures of Subset 2 (mononuclear Ln complexes) from the Cambridge Structural Database over the Ln series.

After the elimination of erroneous entries and those lacking three-dimensional structural information (atomic coordinates), we have found a total of 49472 crystal structures of Ln complexes in CSD (Subset 1). Figure 1 depicts the distribution of Subset 1 across the lanthanide series. The average number of structures available for each Ln element is 3533 (excluding Pm). Subset 2 is a subset of Subset 1 and comprises the 27858 mononuclear Ln complex structures. To make sure that there are no duplicates, each structural entry in Subset 2 (in the MOL format) was converted into a unique hash code (64-bit encoded) using RDKit^35^ and no duplicate hash codes were found. Notably, the number of structures for elements Praseodymium (Pr), Holmium (Ho), Thulium (Tm), and Lutetium (Lu) is smaller than that of other Ln ions.

### Coordination number and first shell distance

**Figure 2:**
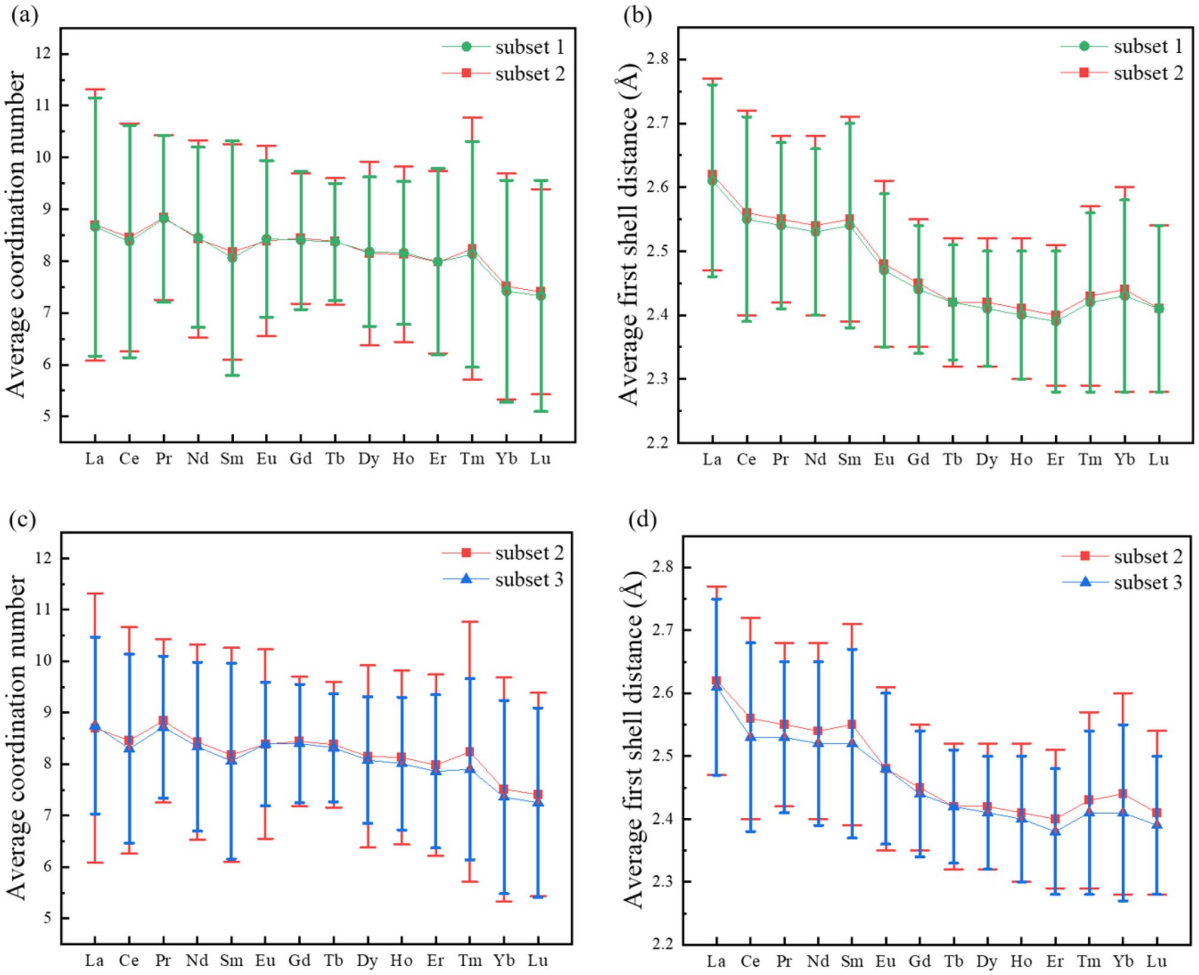
Average coordination number and distance of the first coordination shell of Ln complexes in Subset 1 (green), Subset 2 (red) and Subset 3 (blue; structures in subset 2 but without the cyclopentadienyl ligand; 26,156 structures) across the Ln series: (**a**) Average coordination number for Subsets 1 and 2; (**b**) Average distance of the first coordination shell for Subsets 1 and 2; (**c**) Average coordination number for Subset 2 and 3; (**d**) Average distance of the first coordination shell for Subset 2 and 3. Standard deviations are shown as the error bars. We count the contribution of each cyclopentadienyl ligand to the coordination number as three.

Figure 2a presents the average coordination number (CN) and the corresponding standard deviation for each element. The average CN from La to Lu exhibits a discernible decreasing trend, gradually from 8.66 to 7.33 in Subset 1 and from 8.70 to 7.41 in Subset 2. This is consistent with what has been found in Ln-water complexes of nine-coordinate for light Ln’s and eight-coordinate for heavy Ln’s^36^. The small bumps at Pr and Tm in the overall decreasing trendline might be due to the relatively smaller number of their structures; on the other hand, one also notes that there are large variations in the coordination number for all Ln ions here. Like CN, the average first shell distance (Fig. 2b) also demonstrates a decreasing pattern from La to Lu, decreasing from 2.61 to 2.41 Å for Subset 1 and from 2.62 to 2.41 Å for Subset 2. This decreasing trend in the first shell distance reflects the lanthanide contraction^37^.

**Figure 3:**
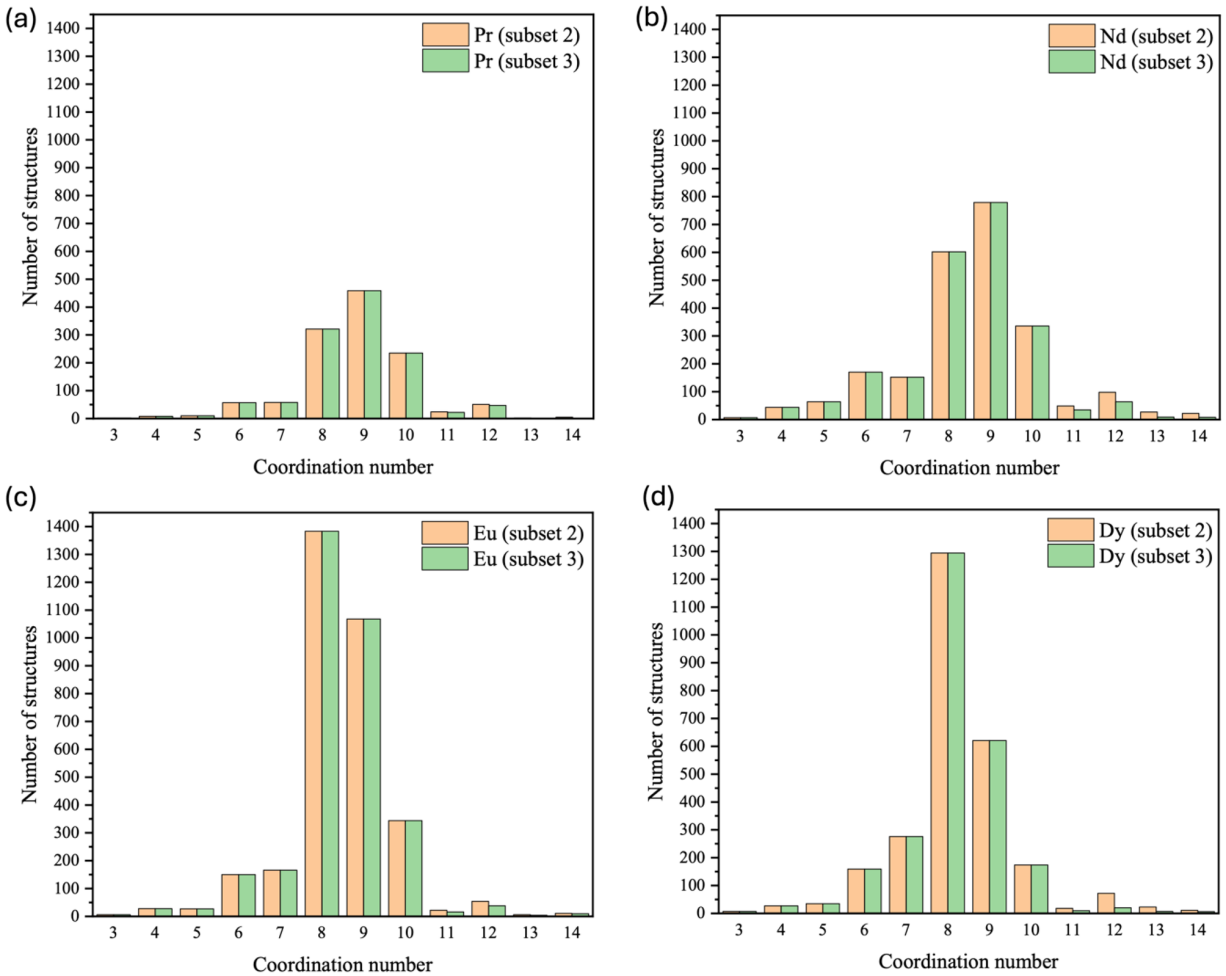
Distribution of the coordination numbers for each Ln ion of structures in Subset 2 and Subset 3: (**a**) Pr; (**b**) Nd; (**c**) Eu; (**d**) Dy. The complete set can be found in Fig. S1 in the SI.

We think that the relatively large deviations in the coordination numbers for Subset 1 and Subset 2 are due to the contribution from the high-hapticity ligands such as cyclopentadienyl (Cp). To test this hypothesis, we created a new subset from Subset 2 by removing structures having the Cp ligand and the resulting subset is called Subset 3. Indeed, one can see that both deviations in the CN (Fig. 2c) and the first shell distance (Fig. 2d) decreases significantly from Subset 2 (with Cp) to Subset 3 (without Cp). We further break down the CN distribution to each Ln ion and selected four representative cases to show in Fig. 3 (the complete set can be seen in Fig. S1). For light Ln ions, CN = 9 is most popular, followed by CN = 8 and then CN = 10 (Fig. 3a,b). Starting with Sm, CN = 8 becomes the most popular, followed by CN = 9 (Fig. 3c,d). Counting in the Cp ligand or out in the structures (that is, comparing Subset 2 and Subset 3 in Fig. 3) has no effect on the distribution of the popular CNs, confirming that it is very reasonable to count each Cp ligand’s contribution to CN as three.

### Donor types, ligand types, and denticities in the complexes

**Figure 4:**
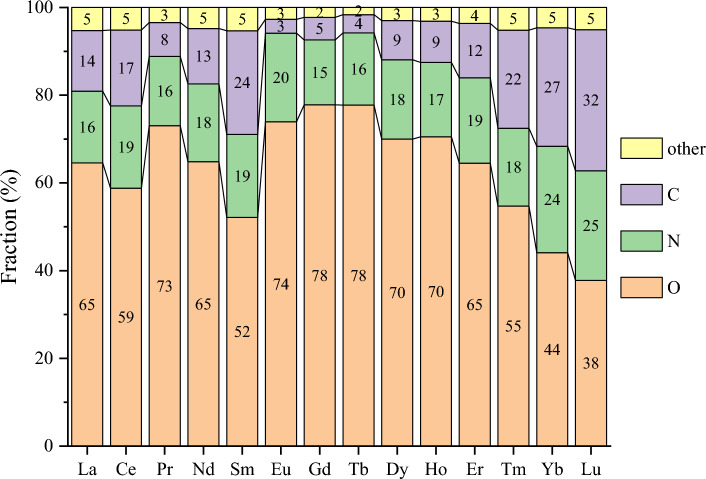
Breakdown of donor types in the first coordination shell for each Ln element in Subset 2. Other elements include H, P, S, F, Cl, Br, and I.

Figure 4 displays the distribution of donor element types in the first coordination shell for each Ln element in Subset 2 (the trend is similar for Subset 1). One can see that oxygen atoms comprise most of the donor groups, followed by carbon atoms and nitrogen atoms. The oxygen donors are 35% inorganic (water and nitrate) and 65% organic (see next section for detailed analysis). The carbon atoms are of mainly the cyclopentadienyl (Cp) ligands, while the nitrogen atoms of mainly the sp^2^ type in an aromatic system (> 70%) such as phenanthroline (see next section for detailed analysis). Collectively, these three elements constitute approximately 95% of the total donor atoms observed within the dataset. Interestingly, one sees more C and N contributions to the first coordination shell in the Yb and Lu complexes. In summary, the predominant presence of oxygen, nitrogen, and carbon atoms as donor atoms highlights their significant role in coordination with lanthanide elements, underlining their prominence in the formation of lanthanide complexes.

**Figure 5:**
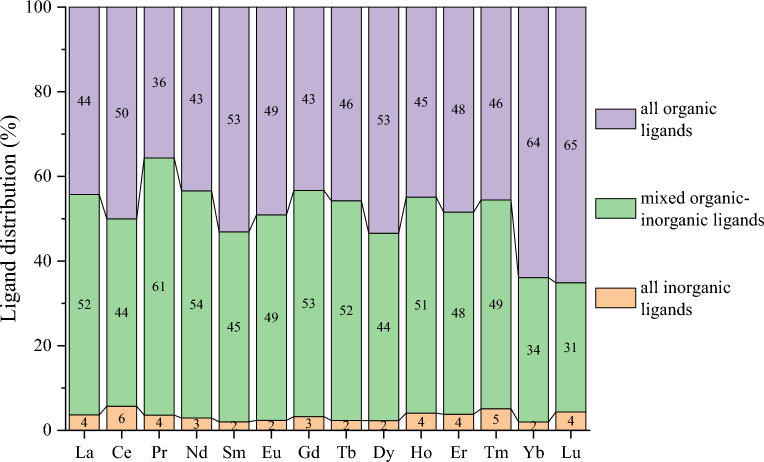
Ligand types in the Ln complexes in Subset 1: complexes with all-organic ligands (purple); complexes with mixed inorganic–organic ligands (green); complexes with all-inorganic ligands (orange).

Figure 5 illustrates the distribution of the type of ligands binding to the metal center of lanthanide complexes in Subset 1. It was found that complexes with all organic ligands account for about 45% from La to Tm and 65% from Yb to Lu of structures, while complexes with mixed organic–inorganic ligands account for 50% from La to Tm and 30% from Yb to Lu of structures. The complexes with all inorganic ligands account for about 5% or less. One can see that the heaviest lanthanides Yb and Lu display dominance of complexes with organic ligands, a trend consistent with the increasing percentage of carbon donors in their first coordination shell (Fig. 4).

**Figure 6:**
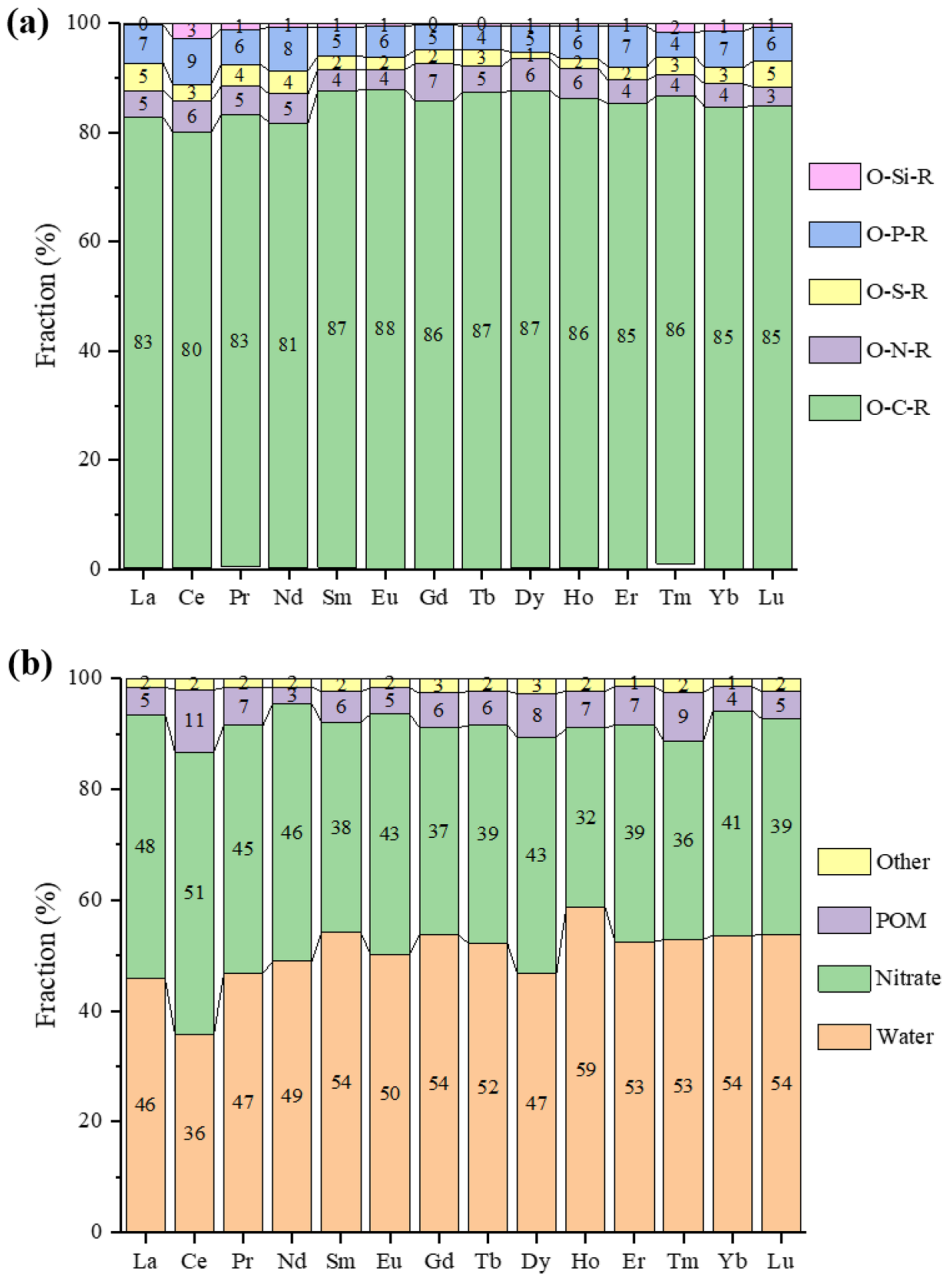
Breakdown of the O donor types in the first coordination shell for each Ln element in Subset 2: (**a**) organic O donors (O-X-R means that an X atom connected to the O donor to Ln); (**b**) inorganic O donors (POM—polyoxometalate).

**Figure 7:**
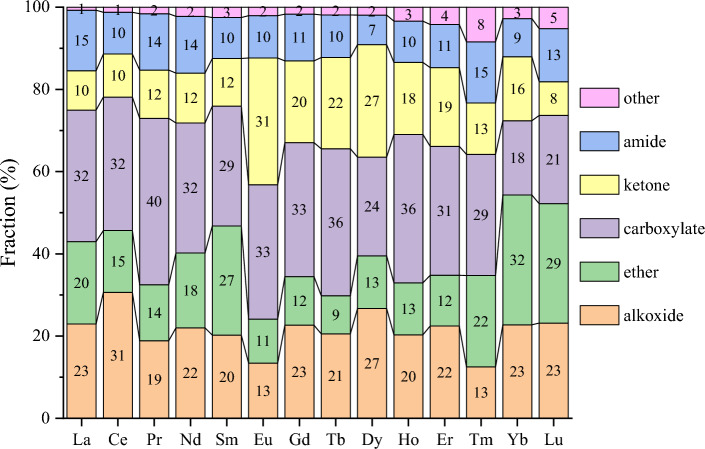
Breakdown of the O-C-R type of ligands in the first coordination shell for each Ln element in Subset 2.

**Figure 8:**
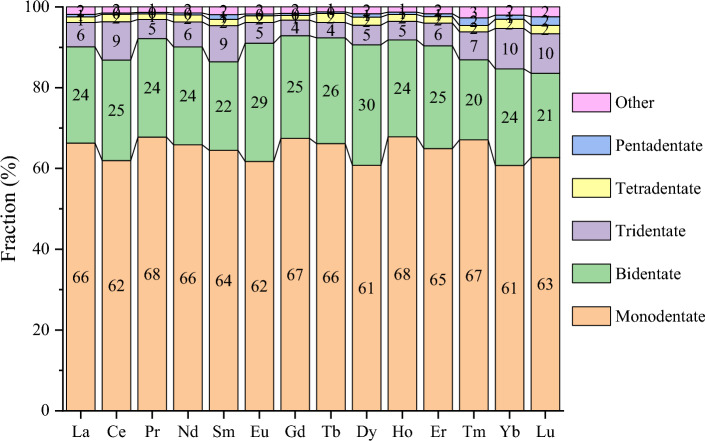
Denticity analysis of ligands in the first coordination shell for each Ln element in Subset 2.

The other way to further examine the ligand types is to breakdown the O donors to functional groups, as the O donors constitute the largest group in the first coordination shell (Fig. 4). We found that among the organic O donors (Fig. 6a), over 80% of them have a C atom connected to the O donor (O-C-R), but there are also some minor contributions of P, N, and S connecting to the O donor. The inorganic O donors (Fig. 6b) are mainly from nitrate and water; there are also minor contributions from polyoxometalate (POM) anions as ligands. We further broke down the O-C-R type of ligands (Fig. 7) and found that alkoxide, ether, carboxylate, ketone, and amide ligands all contribute significantly across the Ln series. In addition, a denticity analysis was conducted on Subset 2 (Fig. 8): one can see that monodentate ligands dominate, followed by bidentate, while contributions from higher denticities are minor. This is consistent with the dominance of water, nitrate, and O-C-R as O donors (Fig. 6).

### Distribution of commercial complexants

**Figure 9:**
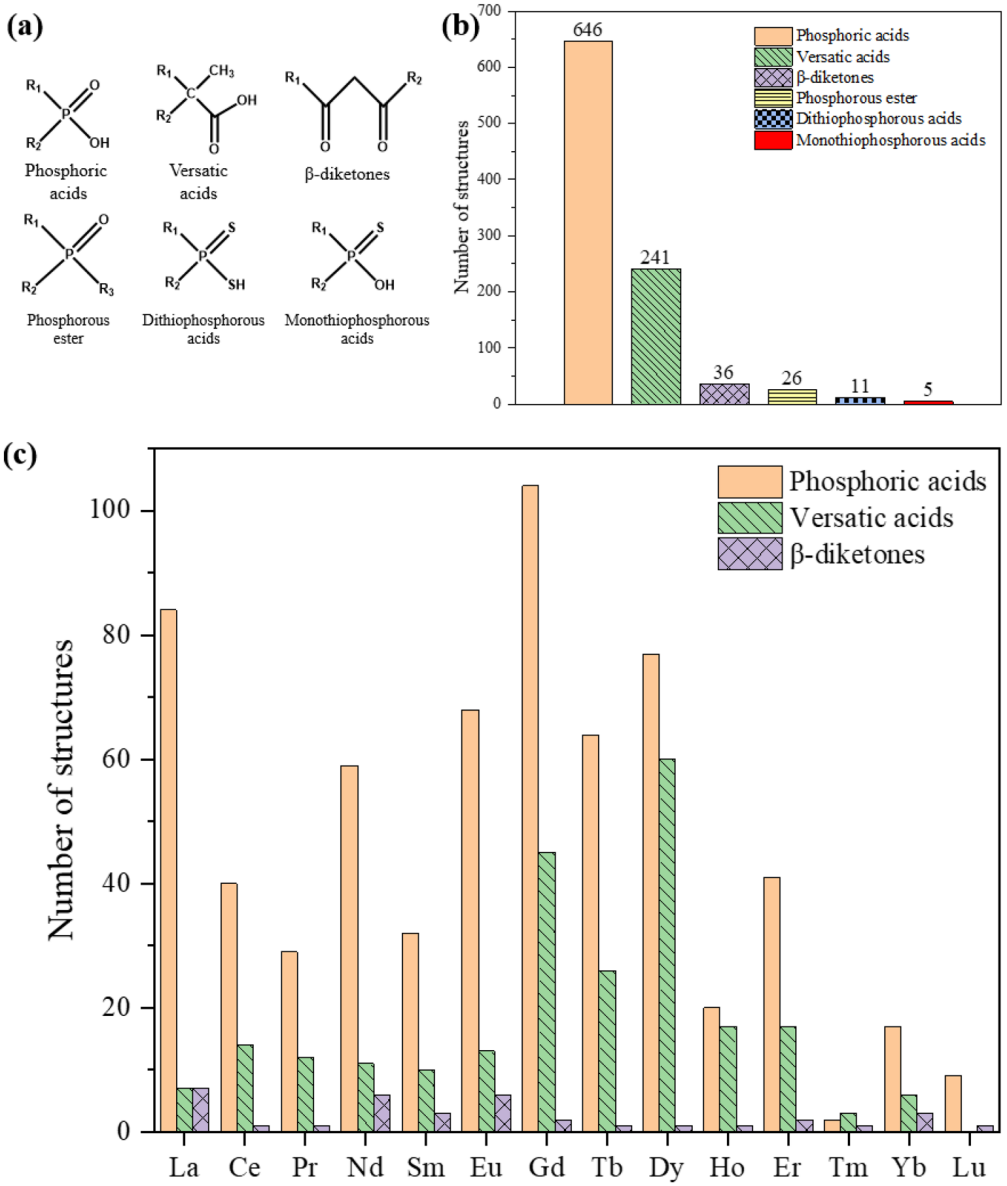
Ln complexes in Subset 1 with selected commercial complexants: (**a**) selected commercial complexants; (**b**) distribution over the ligands; (**c**) distribution across the Ln series (top 3 complexant types).

**Figure 10:**
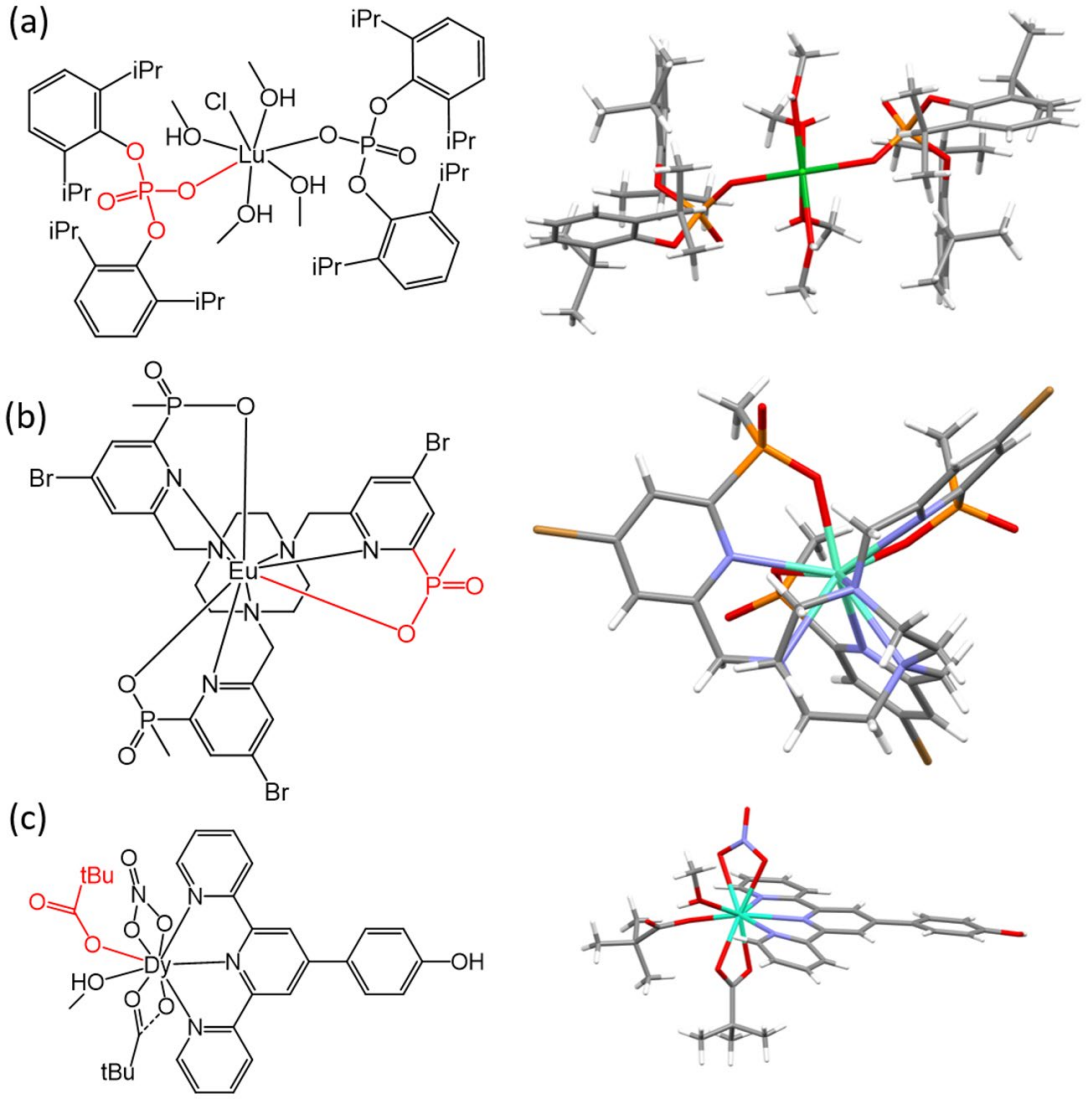
Selected Ln complexes with commercial complexants: (**a**) Lu complex with a phosphoric acid ligand; (**b**) Eu complex with a phosphinic acid ligand; (**c**) Dy complex with a versatic acid ligand.

Since this work is motivated by REE separations, we are especially interested in commercial and state-of-the-art bench-scale ligands and their complexes in CSD. Figure 9a shows selected commercial complexants^4^. A total of 965 complexes were identified, two thirds of which are those of phosphoric acid ligands, followed by one quarter with versatic acids (Fig. 9b). The rest are those of thiophosphorous acids, phosphorous esters, and β-diketones. Breaking down across the Ln series (Fig. 9c), one can see that Gd, La, and Dy have the most structures, while Lu, Yb, and Ho have the least. Figure 10 shows some examples of the Ln complexes with phosphoric acid and versatic acid ligands.

### Phenanthroline and phenanthroline-based ligands

**Figure 11:**
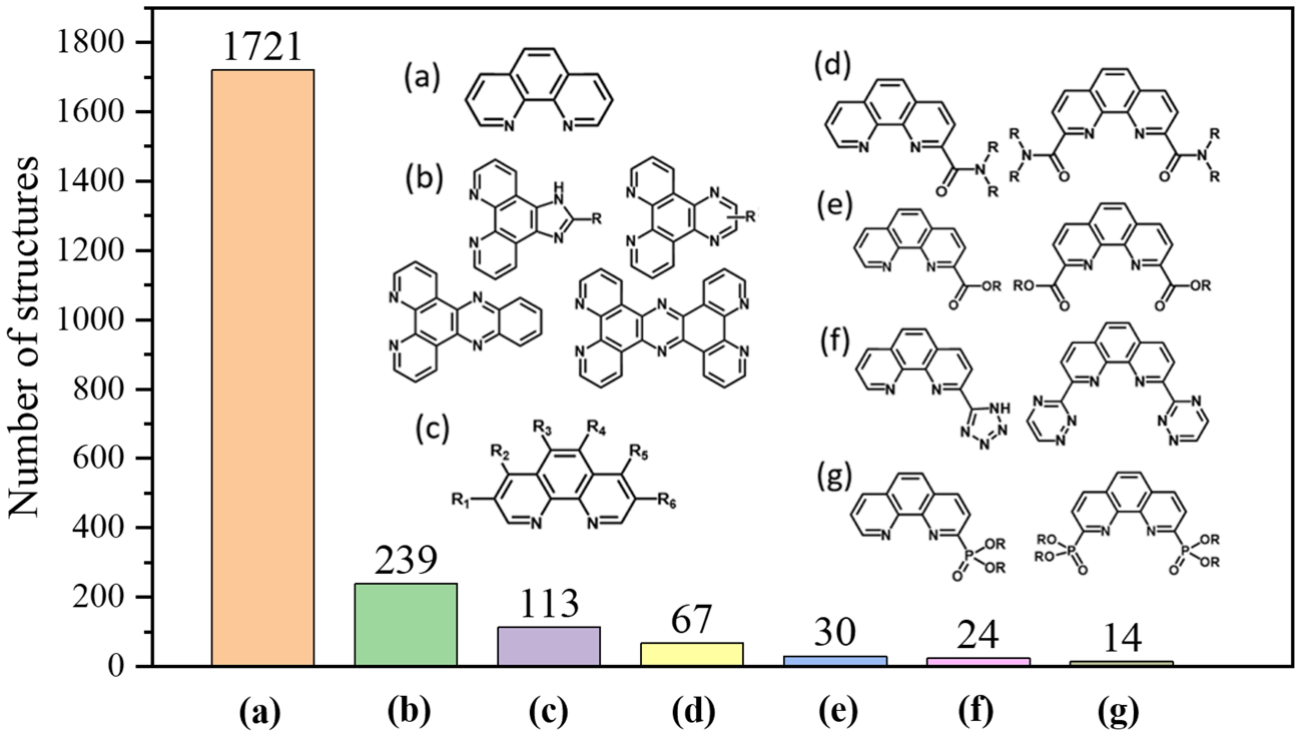
Distribution of Ln complexes in Subset 4 based on the types of the phen and its derivatives: (**a**) phen; (**b**) imidazo/pyrazino; (**c**) substituted phen (R = carbon chain or halogen group); (**d**) amide; (**e**) carboxylate; (**f**) triazine/tetrazole; (**g**) phosphoryl.

**Figure 12:**
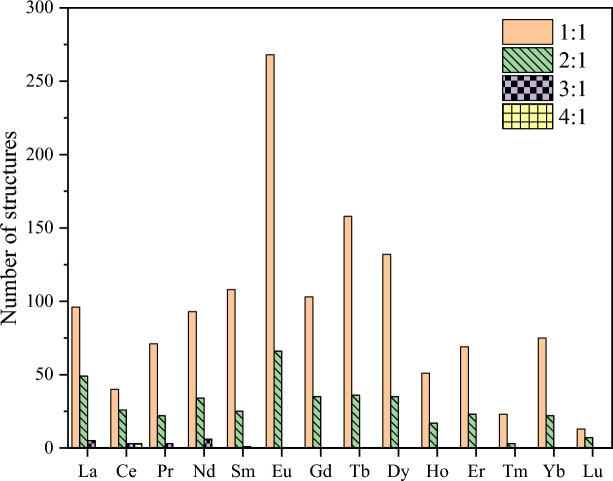
Distribution of the Ln-complex structures in Subset 4 across the Ln series according to the phenanthroline ligand to the metal center ratio.

In examining the complexes with all-organic ligands and mixed inorganic–organic ligands, we have found many Ln complexes with 1,10-phenanthroline (phen) as a ligand. In addition, phen derivatives are actively explored as new ligands for REE separation processes^38^. Subset 4 (2226 structures in total), created from Subset 1, contains Ln-complexes with at least one phen-based ligand. Figure 11 shows the distribution over the phen ligand types within Subset 4: one can see that the majority (1721 structures or 77%) incorporate the phen ligand itself—highlighting its dominance. A distant second is phen derivatives with imidazo/pyrazino groups that extend the conjugation, followed by substituted phen ligands. We further examined the stoichiometry of the phen ligand or its derivative to the Ln center. As can be seen from Fig. 12, the majority of the complexes have 1:1 ligand-to-metal ratio, followed by 2:1; in contrast, 3:1 and 4:1 complexes are rare. This trend is consistent across the Ln series. The dominance of the 1:1 complexes has important implications in designing and employing these ligands for REE separations.

### Accuracy of the Ln-complex datasets based on the CSD structures

Although our analysis above assumed that the structures included in our datasets are accurate and reliable, we acknowledge that there are some inaccuracies in the CSD as documented previously^39,40^. The large variations in the average coordination number and distance of the first coordination shell of Ln complexes seen in Fig. 2 could reflect such inaccuracies causing large noises in the data trendline. On one hand, we think that the average trend should still stand and be useful, assuming that the majority of the structures reported in CSD are good (our analysis of the R-factor indicates that the overwhelming majority of the structures have relatively high quality with R < 0.10, as shown in Fig. S2 in SI); on the other hand, there is a need to use quantum chemical methods such as density functional theory to check and confirm the accuracies of the complex structures in CSD. This will be an important and time-consuming future task to build high-quality structural database for Ln-complexes. In addition, high-throughput automatic structure generation and geometry optimization would accelerate such database-building efforts; the recent work by Yang and coworkers^30^ is an excellent example.

## Conclusion

In this work we have analyzed the currently available structures of lanthanide complexes in the Cambridge Structural Database (CSD) in terms of the coordination shell, donor type, ligand type, denticity, and commercial complexant, from the perspective of rare-earth separations. We found that the average coordination number decreases from 8.7 for La to 7.4 for Lu while the average donor-to-metal distance of the first coordination shell decreases from 2.62 to 2.41 Å. In the first coordination shell, O donors are most popular, followed by C and N donors. There are about 2000 complex structures with commercial complexants, among which phosphoric acid ligands are most popular (66%), followed by versatic acids (25%). Interestingly, 1721 structures incorporate the phen ligand and over 70% of them have 1:1 phen to Ln ratio. These structural insights into lanthanide coordination chemistry will be useful for further data-driven approaches for structure-based design of new ligands for separation of lanthanides.

### Supplementary Information

Supplementary Figures.

## Data Availability

Python scripts used within the CSD Python API and the resulting datasets from CSD associated with the figures in the text can be found in Github (https://github.com/sheinlee/Ln-coordination-insights).
