# Supplementary material for: Insights into coordination and ligand trends of lanthanide complexes from the Cambridge Structural Database
Source: Sci Rep. 2024 May 17;14:11301. doi: 10.1038/s41598-024-62074-3 (PMC11101447; doi:10.1038/s41598-024-62074-3)
Supplement: Supplementary file 1 — Supplementary Figures. [file 41598_2024_62074_MOESM1_ESM.docx]

Supplementary Information

**Insights into coordination and ligand trends of lanthanide complexes from the Cambridge Structural Database**

Shicheng Li,^1^ Santa Jansone-Popova,^2^ and De-en Jiang^1,^*

^1^Department of Chemical and Biomolecular Engineering, Vanderbilt University, Nashville, Tennessee 37235, United States

^2^Chemical Sciences Division, Oak Ridge National Laboratory, Oak Ridge, Tennessee 37831, United States

*To whom correspondence should be addressed. E-mail: de-en.jiang@vanderbilt.edu


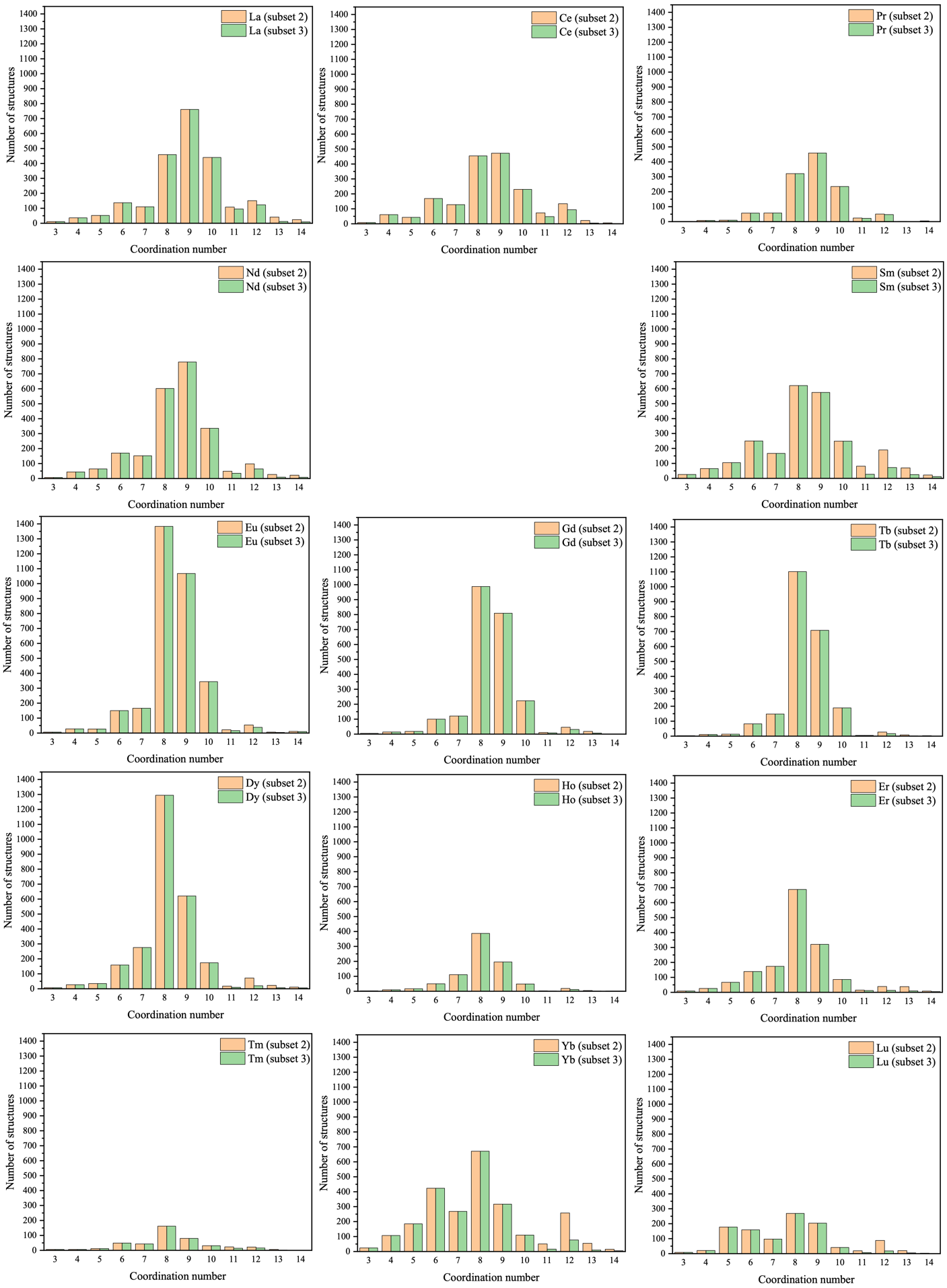


Figure S1. Distribution of the coordination numbers for each Ln ion of structures in Subset 2 and Subset 3.


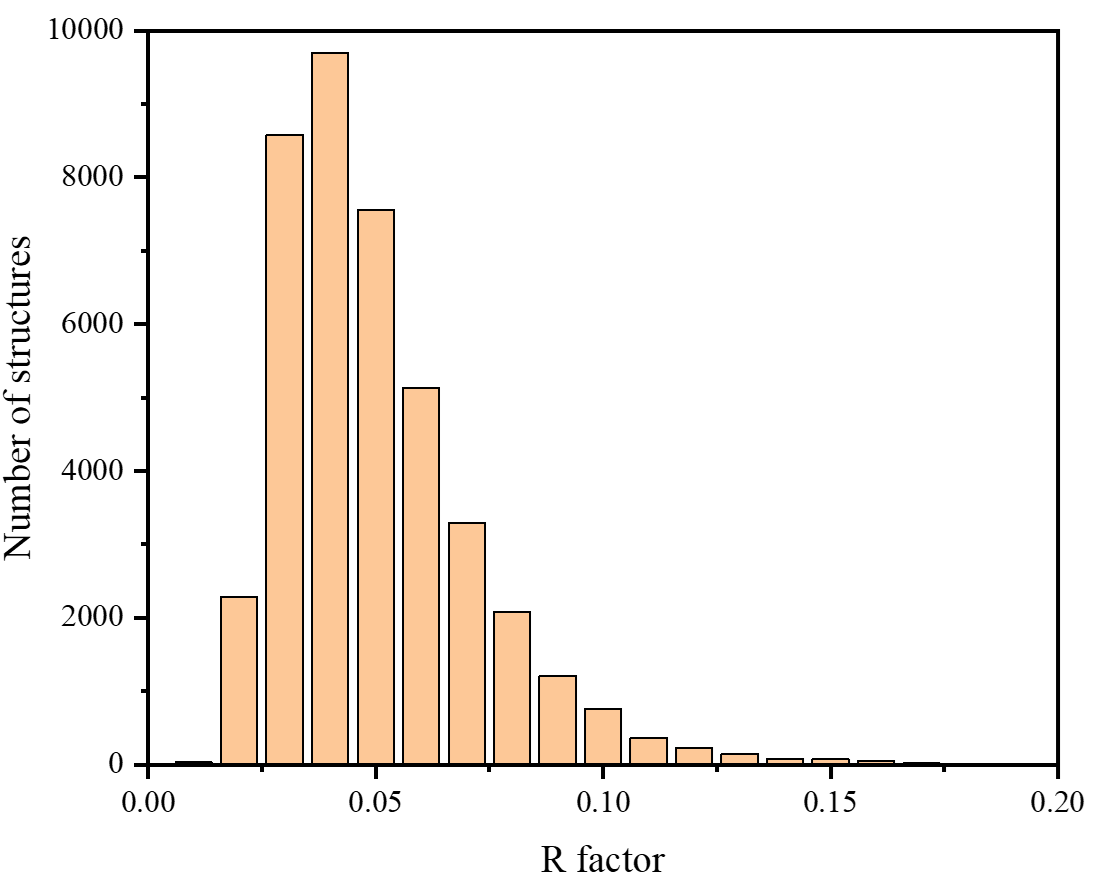


Figure S2. Distribution of R factors for structures in Subset 1.
